# Review of Progress of AI in Biomimetics: From Biological Patterns to Closed-Loop Discovery

**DOI:** 10.3390/biomimetics11050320

**Published:** 2026-05-03

**Authors:** Zhong Hu, Haiping Hong, Tim Lin

**Affiliations:** 1 Department of Mechanical Engineering, J. J. Lohr College of Engineering, South Dakota State University, Brooking, SD 57007, USA; 2 Department of Industrial & Manufacturing Systems Engineering, Iowa State University, Ames, IA 50011, USA; hhong@iastate.edu; 3 Ageis Tech Inc., Santa Ana, CA 92706, USA

**Keywords:** artificial intelligence, machine learning, deep learning, deep reinforcement learning, biomimetic materials, material design and development

## Abstract

Biomimetic materials mimic biological structures and functions. They are crucial for addressing complex challenges in tissue engineering, sustainable architecture, and energy storage. Traditionally, designing these materials requires slow, resource-intensive trial-and-error methods and physics-based simulations. Recently, Artificial Intelligence (AI) and Machine Learning (ML) have transformed this field. They translate biological intelligence into actionable engineering logic and rapidly explore massive design spaces. Despite rapid advancements, the field still faces several critical bottlenecks, including complexity mismatches, data scarcity, and limited interpretability. This review examines AI-driven biomimetic design across five primary “interfaces”: (1) Biological Pattern Recognition, (2) Structural Optimization, (3) Generative Morphogenesis, (4) Adaptive Fabrication, and (5) Data-Driven Discovery Platforms. The review also outlines future perspectives, especially the shift toward autonomous “closed-loop” laboratories. In these labs, AI will manage the entire workflow, i.e., design, synthesis, and testing, without human intervention. Future efforts will likely focus on multi-model data mining to understand complex, life-like properties. Furthermore, research aims to develop Explainable AI (XAI) to ensure deterministic modeling in safety-critical applications. The ultimate goal is a synergistic relationship. AI will design materials, but these materials, using biomimetic metabolic or neural models, will also help construct more efficient AI architectures.

## 1. Introduction

For over 3.8 billion years, nature has refined materials with extraordinary synergy between form and function, often surpassing synthetic counterparts [1,2]. Consequently, biomimetic engineering has become a cornerstone of innovation, providing transformative solutions for challenges in regenerative medicine, urban sustainability, and energy storage [2].

Historically, the development of biomimetic materials has followed a “biological extraction” path: researchers first identified a biological feature, subsequently analyzed it via microscopic observation or mechanical testing, and then attempted replication through iterative trial-and-error. While this approach has successfully yielded landmark innovations, such as Velcro or gecko-inspired adhesives, it faces significant bottlenecks [3]. Physics-based simulations, such as Computational Fluid Dynamics (CFD), Finite Element Analysis (FEA), Molecular Dynamics (MD), or Density Functional Theory (DFT), offer high precision but incur substantial computational costs. These techniques struggle to scale effectively when exploring the multi-dimensional design spaces for hierarchical biological structures [3]. The complexity inherent in living systems arises from cross-scale interactions, extending from the molecular level to specific industrial applications. Understanding these interactions is critical for advancing biomimetic materials, but bridging the gap between biological complexity and engineering application remains a challenge.

The integration of Artificial Intelligence (AI) and Machine Learning (ML) has shifted the paradigm of materials science from manual parameter tuning to interpreting the biological intelligence embedded in natural systems [4]. By framing materials design as a generative or optimization problem, AI algorithms, specifically deep learning (DL) and deep reinforcement learning (DRL), can explore millions of potential configurations, reducing the discovery cycle from years to weeks. The true significance lies in the capacity to design de novo biomimetic materials. These materials do not simply copy nature but optimize their principles for specific industrial applications.

Despite this momentum, several critical gaps obstruct the transition from bio-inspiration to functional synthetic materials. Biological materials are inherently hierarchical and multi-functional. For example, bone provides mechanical support while simultaneously facilitating metabolic processes and maintaining mineral homeostasis [5]. Current AI models are typically optimized for single-objective tasks (e.g., maximizing stiffness or conductivity) and therefore struggle to replicate the intricate, multi-functional characteristics of biological tissues. AI effectiveness depends heavily on high-quality, annotated data, which is often scarce, sparse, or noisy in materials engineering compared to other fields. A major bottleneck is translating AI-predicted designs into physically manufacturable materials. Many models focus on functionality but lack insight into whether a material can actually be synthesized.

The second major hurdle lies in the scarcity and quality of data. Unlike the fields of computer vision (CV) or natural language processing (NLP), biomimetic datasets are fragmented, costly to produce, and biased by a lack of reported negative results [6]. Furthermore, the “black box” nature of advanced neural networks limits their use in safety-critical sectors like aerospace, where regulatory approval requires explainable AI (XAI) [7]. Additionally, AI-generated hierarchical structures often surpass current additive manufacturing (AM) capabilities, creating a gap between design and physical production.

The current research landscape is rapidly merging AI and biomimetics across five core “interfaces”. First, convolutional neural networks (CNNs) have streamlined biological pattern recognition, enabling high-throughput (HT) digitization of nature’s designs [8,9,10,11,12,13,14,15,16,17,18,19,20,21]. Second, structural optimization has shifted toward a “materials by design” paradigm, using ML models replacing costly simulations for real-time property predictions [22,23,24,25,26,27,28,29,30,31,32,33,34,35,36,37,38,39]. Third, generative morphogenesis uses Generative Adversarial Networks (GANs) and Variational Autoencoders (VAEs) to create novel architectures that maintain biological characteristics while exploring non-natural geometries [40,41,42,43,44]. Fourth, adaptive fabrication technologies integrate AI with 3D and 4D printing through machine vision systems that monitor and correct defects in real-time [45,46,47,48,49,50,51,52,53,54]. Finally, data-driven discovery platforms unify experimental and simulation data to accelerate AI-driven material exploration [9,55,56,57,58].

**Figure 1:**
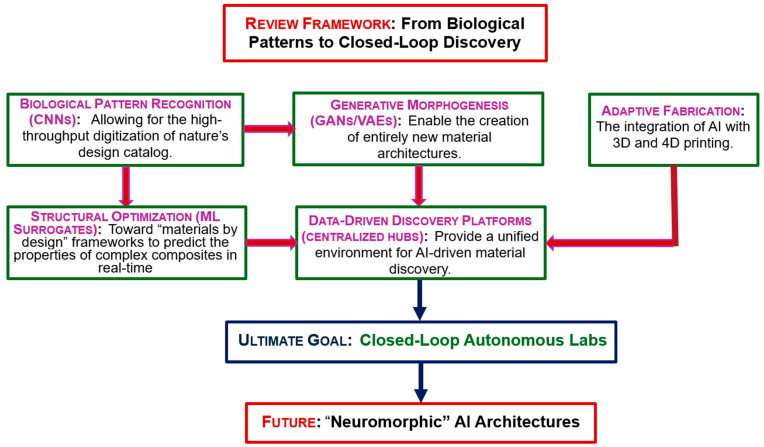
Schematic flowchart of the review framework.

While prior reviews have focused heavily on individual machine learning applications in material characterization. This review addresses the critical gap regarding the systemic integration of autonomous, multi-agent AI systems that bridge the entire, non-interventional closed-loop from biological pattern mining to robotic synthesis. This review examines key breakthroughs driving us toward fully autonomous, “closed-loop” laboratories. Within these facilities, AI runs the entire research pipeline. It autonomously handles biological data mining, robotic synthesis, material testing and fabrication, without human intervention. We envision a mutually beneficial future for humans and matters. By using biomimetic, metabolic, or neural models, future materials will enable more efficient “neuromorphic” AI architectures. Figure 1 illustrates this review workflow.

## 2. Biological Pattern Recognition: Digitizing Nature’s Design Catalog

Biological systems are the products of millions of years of evolutionary optimization. They feature highly complex engineered structures, ranging from spiral-reinforced bones to the interlocking, hierarchical architectures of nacre. A deep understanding of these complex hierarchies is the cornerstone of biomimetic material research. For decades, the field was limited to a manual observation paradigm. Traditionally, identifying these structures requires tedious, qualitative, and subjective analysis of biological images acquired via scanning electron microscopy (SEM), transmission electron microscopy (TEM), or computed tomography (CT). High-resolution imaging techniques, such as micro-CT (µCT), cryo-electron microscopy, and confocal imaging, have created a “big data” challenge. The enormous volume of information generated far exceeds human capacity to analyze and annotate it. Consequently, biological pattern recognition has shifted from a descriptive discipline to a computational one. The rise of AI, especially CNNs and DL techniques, has revolutionized this process. These AI tools enable automated, HT, and objective quantitative analysis of structural motifs. This shift transforms analog biological images into digitized “design catalogs”. It provides data-driven support for generative design and biomimetic fabrication [8,9,10,11,12,13,14,15,16,17,18,19,20,21].

### 2.1. The Shift from Manual Feature Engineering to Deep Learning

Historically, interpretation of biological structures relied on manual feature engineering. Experts quantified parameters, such as porosity, trabecular pore size, nacre aspect ratio, or fiber orientation, from μCT or SEM images, using traditional CV methods like Sobel filters or Watershed segmentation. While effective for simple geometries, this approach is labor-intensive, subjective, and struggles with the inherent biological variability. Crucially, it fails to capture the complex, multi-scale, and nonlinear interactions that determine the performance of natural materials, such as hierarchical nacre or porous bone [59,60].

**Figure 2:**
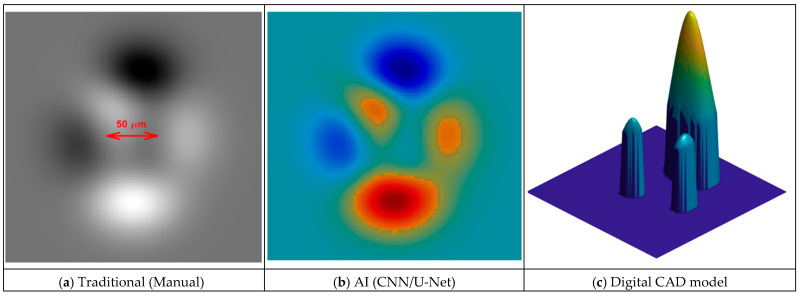
Schematic of the evolution in biological pattern recognition.

AI has overcome the bottlenecks of manual analysis through automated feature extraction [61]. Using bio-inspired, hierarchical architectures, CNNs accurately identify complex structural patterns [62], a shift illustrated in Figure 2 [63,64,65,66,67,68]. Figure 2a depicts a standard SEM image of a biomaterial, e.g., bone, where red arrow indicates antiquated manual spot-checking, such as hand-measuring a 50 μm pore. In contrast, Figure 2b shows the same image processed by a U-shaped Convolutional Network (U-Net). The resulting color-coded semantic segmentation mask demonstrates how CNN automatically delineates intricate trabecular structures, replacing manual effort. Finally, Figure 2c shows these CNN-optimized structures converted into a CAD-compatible 3D model. This 2D-to-3D transformation enables direct export for 3D printing or FEA simulation, moving beyond simple visual inspection toward automated, quantitative characterization.

The advantages of employing AI techniques for automated feature engineering can be summarized in three main aspects [69,70,71,72,73,74,75,76].

- **Automation of Feature Extraction**: CNNs can automatically identify structural motifs, such as seashell spirals or bone trabeculae, without manual specifications. Through layered convolutional operations, these networks recognize edges, textures, and complex geometries, eliminating tedious manual feature engineering. Lower layers detect basic units (like mineralized fibrils), while deeper layers synthesize them into complex arrangements, such as Bouligand structures. Additionally, algorithms like U-Net or Mask R-CNN automate the isolation and quantification of components within complex biological images.
- **HT Digitization**: AI-driven segmentation and feature extraction digitize biological image datasets faster and more objectively than human experts. For example, automated image analysis of trabecular bone structures rapidly quantifies density, orientation, and connectivity. This data then can be directly fed into FEA models to optimize the design of lightweight materials.
- **Contextual Understanding**: The transition to DL techniques marks a shift from traditional *top-down*, *rule-based* research to *bottom-up*, autonomous exploration. Unlike traditional algorithms, CNNs eliminate the need for pre-defined features by using backpropagation to autonomously extract “design rules” through a hierarchical filter system.

Initial layers identify simple features like edges, while deeper layers synthesize them into complex structural motifs, such as the helical pitch of cellulose nanocrystals or the gradient pores in bamboo. For example, training a CNN on thousands of avian feather barbs or butterfly wing scales allows researchers to quantify the spatial frequencies driving structural coloration or aerodynamic lift. By digitizing qualitative biological observations into quantitative “tensor”, these models capture multi-scale spatial hierarchies for direct use in material optimization workflows.

### 2.2. Automated Motif Extraction and Morphological Mapping

AI-driven biomimetic material design converts complex and disordered biological data into functional engineering principles. This process involves two key steps: (1) Automated Motif Extraction, identifying functional, recurring substructures, and (2) Morphological Mapping, organizing these motifs within a searchable, multidimensional “morphological space”. Unlike traditional, subjective manual analysis, AI/ML enables rapid and unbiased identification of design rules, transforming material engineering from empirical trial-and-error to data-driven design [77,78,79,80].

AI routinely employs CNNs, such as U-Net or ResNet, to segment complex biological composites, identifying structural motifs essential for biological function. In bone tissue engineering, these models map the osteon patterns and porosity to elucidate how biological structures adapt to local stress, providing blueprints for 3D-printed scaffolds that mimic natural mechanical anisotropy. To overcome data scarcity, transfer learning allows high-accuracy recognition using limited biological samples, with networks pre-trained on massive datasets (e.g., ImageNet) and fine-tuned for specific biological microscopic images. This approach accelerates the characterization of rare biological species and expands the repertoire of nature-inspired, AI-driven material solutions [81,82,83].

#### 2.2.1. Automated Motif Extraction

**Figure 3:**
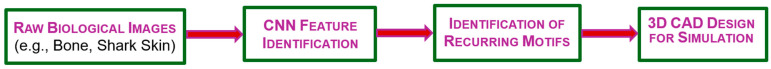
Schematic diagram of the AI-driven motif extraction workflow.

Automated motif extraction techniques integrate advanced CV and NLP techniques to identify recurring patterns within high-resolution images (e.g., SEM, TEM, CT scans). CNNs efficiently scan 2D or 3D datasets using learnable kernels, capturing hierarchical biological structures, such as porous networks, interlocking textures, or complex protein arrangements. Figure 3 illustrates the AI-driven workflow for the motif extraction: (1) Input: raw biological images (e.g., bone, shark skin) are fed into the system; (2) Feature identification: CNNs analyze the images to identify recurring features; (3) Extraction and Translation: The detected motifs are converted into 3D CAD models; and (4) which are then used for simulation [84,85,86].

- **Image-Based Motif Extraction:** CNNs trained on biological imaging datasets (e.g., bone microstructure, lotus leaf surfaces, and nacreous layers) automate the segmentation, classification, and analysis of structural motifs. The “Center-Environment Segmentation” (CES) model improves upon traditional edge detection by capturing complex textures while accounting for surrounding context [86,87,88].
- **Text/Data-Driven Extraction:** NLP techniques, particularly Transformers and Large Language Models (LLMs), mine millions of biological research papers to extract specific structural parameters (e.g., the stiffness of collagen). Tools like NanoSafari use these algorithms to identify optimal nanoparticle characteristics from literature [89].

#### 2.2.2. Morphological Mapping and Morphological Space Generation

**Figure 4:**
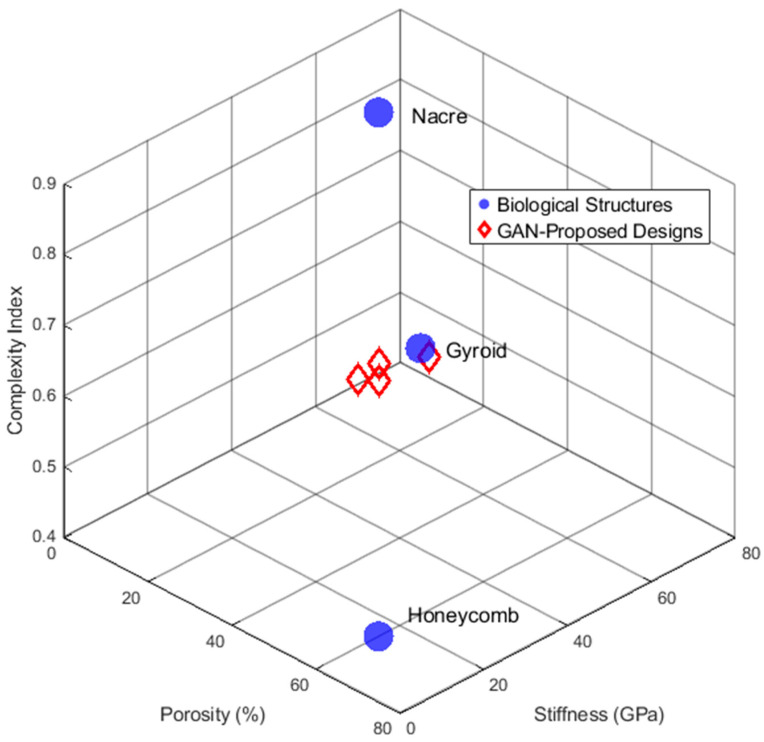
Schematic illustration of biomaterial morphological mapping.

Extracted functional motifs are mapped into a high-dimentional structured “morphological space”, defining materials by their features, such as stiffness, porosity, or interlocking geometries, rather than composition. This representation allows AI to analyze topological relationships, with GANs fillingvacant spaces to generate novel designs (Figure 4) [79,90,91,92,93,94,95,96,97,98,99,100,101,102,103,104,105].

- **Dimensionality Reduction:** Techniques like Principal Component Analysis (PCA) and t-distributed Stochastic Neighbor Embedding (t-SNE) project complex, high-dimensional biological data into a lower dimensional space to visualize and analyze the evolutionary morphology trajectories.
- **GANs and VANs:** These DL models map natural structures to create synthetic motifs, filling morphological gaps to bridge nature-inspired design and material synthesis.
- **Predictive Morphology:** Emerging AI models correlate structural features directly with molecular data (e.g., transcriptomic data), enabling researchers to predict cellular responses to biomimetic scaffolds.

### 2.3. HT Digitization and Design Space

Integrating CNNs with high-speed imaging techniques enables the construction of Digital Morphological Libraries, bridging the gap between biological intelligence and actionable engineering logic. By analyzing biological structures, such as impact-resistant pomelo honeycomb structures, CNNs extract quantitative data, including wall thickness, cell diameter, and vertex angles. This digitization process digitizes nature, initiating a closed-loop design cycle where biological patterns are simulated and iterated for enhanced design.

**Figure 5:**
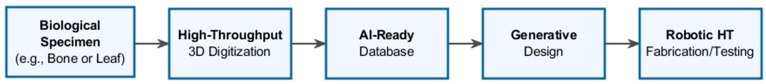
Schematic of industrial digital infrastructure: HT digitization and design workflow.

This approach shifts biomimetic design from manual imitation to a precise, industrial-scale methodology, using high-resolution techniques, e.g., μCT/SEM, to create AI-ready datasets [91,92,93,94,95,96,97,98,99,100,101,102]. As depicted in Figure 5, the workflow follows a precise sequence: biological sampling, HT 3D digitization, AI-ready database creation, generative design, and robotic HT fabrication and testing.

#### 2.3.1. Multi-Dimensional Digitization of Biological Templates

Researchers use advanced imaging and phenomics to create digital models of biological specimens for engineering. HT synchrotron µCT technique, optimized by autonomous robotic scanning systems, enables non-destructive, multi-scale anatomical preservation. This process captures data ranging from sub-wavelength light-modulating structures to hierarchical bone porosity. These data populate AI-ready databases like the Materials Project (MP) to train generative models for novel structural designs.

- **3D Anatomical Preservations**: HT synchrotron µCT technique non-destructively captures 3D structures, with robotic systems automating sample positioning to optimize scanning efficiency.
- **Quantitative Phenomics**: Digitization transcends basic morphology, acquiring multi-scale data from sub-wavelength optical structures to complex cancellous bone hierarchies.
- **Biomimetic Databases**: Curated resources, such as the MP or the Block Copolymer Phase Behavior Database (BCDB), provide the foundation for training generative models to replicate biological structural motifs.

#### 2.3.2. HT Experimental Platforms

HT experimental platforms accelerate digital design exploration by enabling simultaneous testing of thousands of material variants. These systems combine microarrays for combinatorial testing, microfluidics for precise environmental control, and 3D printing for rapid fabrication of biomimetic structures.

- **HT Experimental Platforms**: Enable parallel screening of thousands of material variants, accelerating data generation for digital design templates.
- **2D and 3D Microarrays**: Rapidly deposits polymers, proteins, or cells onto surfaces or encapsulates them in hydrogel, enabling combinatorial analysis of multiple factors, such as stiffness, geometry, and biochemical cues, in a single experiment.
- **Microfluidic Integration**: Provides precise control over environments, allowing parallel screening of drug responses or cellular migration while minimizing reagent use.
- **3D Tissue Models**: Advanced fabrication techniques, such as projection micro-stereolithography and digital light processing (PµSL/DLP), can rapidly construct complex, biomimetic 3D structures ranging from the micro- to nano-scale.

#### 2.3.3. AI-Driven Design Space Navigation

AI accelerates biomimetic design by navigating vast, high-dimensional spaces of material composition, geometry, and fabrication parameters, automating workflows from predictive modeling to data management. The key capabilities include:

- **Autonomous Optimization**: Bayesian Optimization and Active Learning directly integrate with synthetic hardware to autonomously determine optimal conditions, such as inkjet droplet deposition, eliminating human intervention.
- **Rapid Materials Discovery**: Robotic “self-driving labs” (SDLs) combine AI modeling with automated testing, accelerating new materials discovery up to 370 times faster than manual approaches.
- **Predictive and Generative Modeling**: Platforms like MatCloud utilize quantum mechanics and ML to predict material properties, providing a fully automated, end-to-end solution.

### 2.4. Digital Morphological Library Example

To address the need for structural data, MP functions as a crucial digital morphological library, providing extensive computational data on crystal structures. These data enables AI to identify recurring grometric and topological patterns in biomineralized structures, such as nacre or sea urchin spines. Furthermore, specialized databases, including AskNature and bone microstructure databases, are being converted into structured formats for neural network training [106,107,108].

To overcome limited labeled data in biomimetics, transfer learning allows researchers to fine tune pre-trained models (e.g., from ImageNet) using small and specialized datasets. This technique enables effective analysis of sparse biological images, such as SEM images of lotus leaves or butterfly wings [109,110,111].

### 2.5. Limitations and Challenges

While AI-driven biological pattern recognition enables HT digitization of natural designs, it faces significant challenges: scarce large-scale annotated datasets, high-noise data, complex data management, and limited model generalization. Furthermore, translating these digitized, hierarchical motifs into functional, producible designs is a critical bottleneck, compounded by the need for better interdisciplinary collaboration. Finally, the multi-scale complexity of biological structures often causes crucial, subtle features to be overlooked, while the “black box” nature of CNNs limits the interpretability necessary for engineering applications [112,113,114,115,116,117].

## 3. Structural Optimization: Bridging Form and Function

Translating biological design rules into synthetic materials has historically been a slow, arduous, and resource-intensive process. Traditional methods, relying on manual observation, trial-and-error, and conventional physics-based simulations, such as CFD, FEA or MD, struggle to model the hierarchical, nonlinear, and multi-scale nature of biological systems. While structural optimization, such as topology optimization (TO), is crucial for tuning natural motifs into high-performance architectures, conventional approaches struggle to handle these complex, multi-scale, and nonlinear constraints [22,23,24,25,26,27,28,29,30,31,32,33,34,35,36,37,38,39,118,119,120,121]. AI has revolutionized this phase by augmenting iterative solvers with predictive models, enabling rapid navigation of high-dimensional design spaces with unprecedented speed.

### 3.1. Machine Learning-Accelerated Topology Optimization

**Figure 6:**
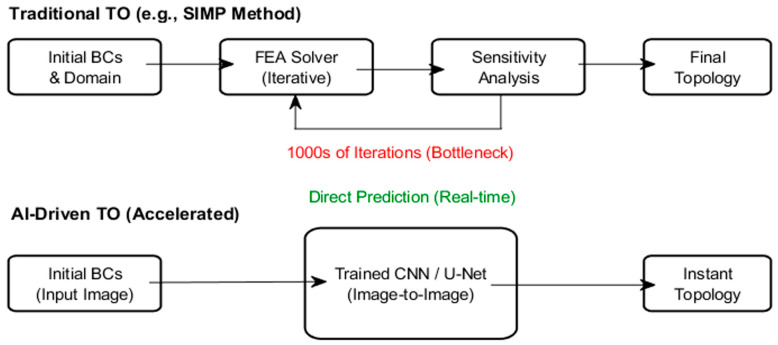
Schematic diagram comparing traditional TO with AI-driven TO.

Traditional TO methods, such as Solid Isotropic Material with Penalization (SIMP) method, rely on slow, iterative FEA loops. In contrast, DL models, such as CNNs and U-Nets (Figure 6), accelerate this process by predicting optimized topologies directly from initial boundary conditions [122]. By treating TO as an image-to-image transformation, AI sidesteps the FEA computational bottleneck to enable real-time, high-fidelity design generation, such as complex biomimetic lattices. Unlike the traditional iterative loop, AI provides nearly instant, feed-forward predictions, significantly reducing the time needed for design optimization [123].

### 3.2. Multi-Objective and Multi-Scale Optimization

Biomimetic materials often require balancing conflicting objectives, such as optimizing strength against density or thermal insulation against structural integrity. DRL is a powerful tool for this multi-objective structural optimization, as agents actively explore the design space to maximize rewards for meeting specific mechanical targets. For instance, DRL optimizes brick-and-mortar microstructures to mimic the crack-deflection toughening mechanisms found in mollusk shells [40].

**Figure 7:**
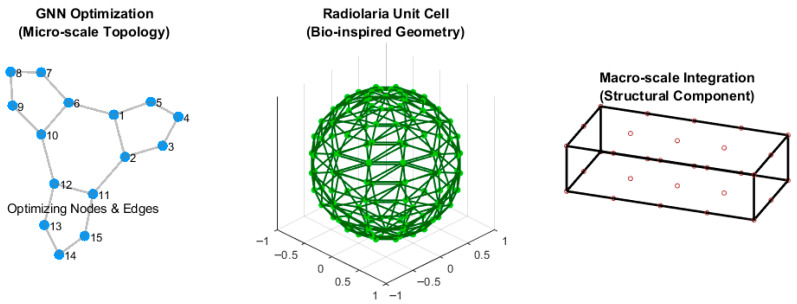
Schematic of a multi-objective and multi-scale biomimetic optimization workflow.

Additionally, Graph Neural Networks (GNNs) are increasingly used to optimize discrete structural systems, such as truss-based metamaterials. By representing materials as nodes (atoms or joints) and edges (bonds or struts), GNNs efficiently optimize the connectivity and geometry of lattice structures to mimic the energy-absorption capabilities of honeycomb or bamboo architectures [124]. Figure 7 illustrates this multi-objective, multi-scale biomimetic optimization workflow, showing a GNN-optimized radiolarian-inspired micro-lattice, its 3D unit cell, and its final structural beam integration.

### 3.3. Surrogate Models for High-Fidelity Simulations

Biomimetic optimization faces a major obstacle: the complexity mismatch between simplified computational models and the intricate reality of biological tissues. To overcome this, AI-driven surrogate models, such as Gaussian Processes or Multi-Layer Perceptrons, approximate complex system responses, acting as “digital twins” for physical materials that enable millions of rapid, cost-effective virtual tests. Recent studies have utilized these models to optimize 3D-printed biomimetic scaffold geometries, allowing precise tuning of pore sizes and interconnectivity for enhanced cell signaling and nutrient transport [125].

**Figure 8:**
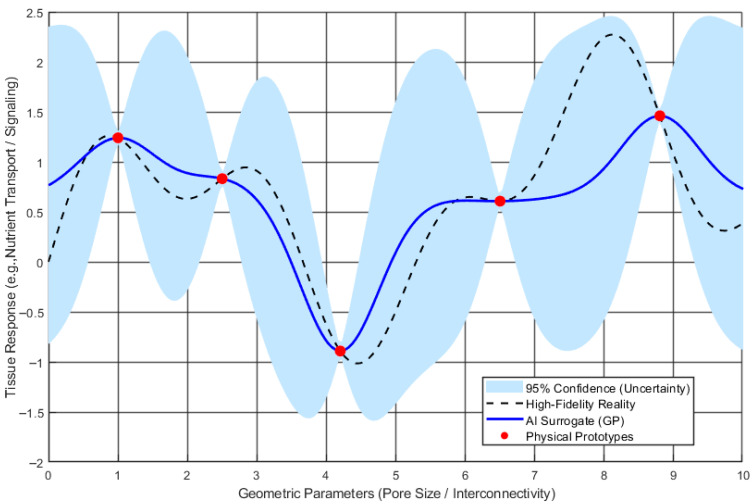
Schematic of an AI surrogate modeling for bridging the complexity mismatch.

Figure 8 illustrates how AI surrogate modeling bridges the complexity mismatch between expensive high-fidelity real-world data and simulation requirements, using a Gaussian Process to map intricate biological tissue responses. It contrasts sparse, costly high-fidelity physical data (Red Dots) with a continuous approximation curve, enabling rapid virtual tests. The figure displays the high-fidelity reality (Dashed Line) via a Gaussian Process output (Solid Blue Line), with the shaded region (confidence interval) highlighting prediction uncertainty and guiding further physical sampling. Acting as a digital twin, AI-driven surrogate model facilitates efficient biomimetic optimization by replacing expensive simulations with accurate approximations [126,127,128,129,130].

**Figure 9:**
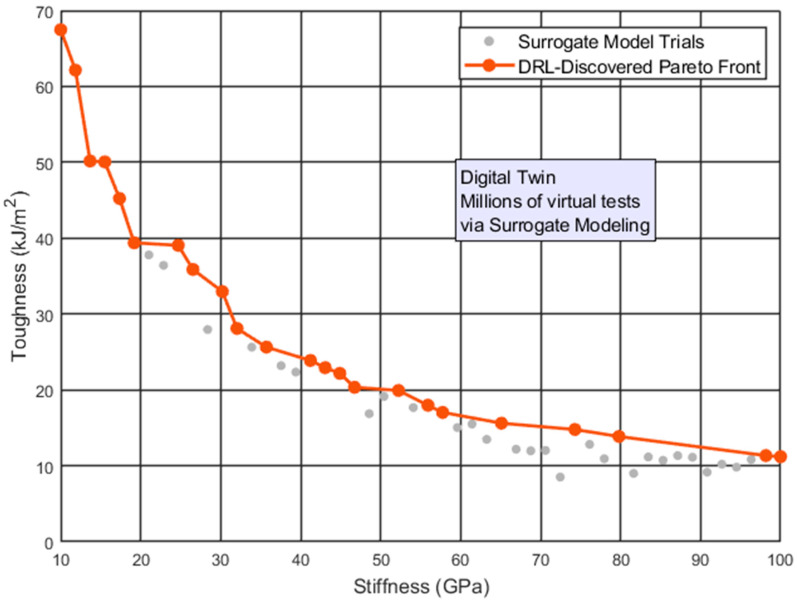
Pareto Fronts in DRL: navigating mechanical trade-offs in nacre-inspired composites.

Figure 9 displays a synthetic Pareto front representing nacre-inspired composites, illustrating the trade-offs where increasing stiffness reduces toughness. A DRL agent navigates this conflict, with the orange curve tracking its path toward an optimal balance by tuning geometric parameters like lamellae aspect ratio. Gray points represent surrogate model tests (Gaussian Process/MLP) that bypass computationally prohibitive simulations.

### 3.4. Quantitative Comparison: Traditional vs. AI-Driven Topology Optimization

AI-driven TO transforms the design process by cutting computational time by up to 30× compared to traditional simulation-heavy methods (e.g., CFD/FEA). Instead of slow, iterative physics-based simulations, AI employs predictive surrogate models for near-instant exploration of complex high-dimensional design spaces. A quantitative comparison is provided in Table 1 [41,131,132,133,134,135].

### 3.5. Limitations and Challenges

Traditional structural optimization methods in biomimetics face significant challenges, primarily due to the high computational costs, specifically when modeling hierarchical multi-scale structures and nonlinear, large-deformation behaviors. Furthermore, these approaches struggle to integrate complex, optimized geometries with practical fabrication constraints. When employing AI, the process often relies on scarce high-fidelity simulation data to bridge the gap between biological inspiration and synthetic production [136,137,138,139,140,141,142,143].

## 4. Generative Morphogenesis: Beyond Biomimetics Toward Non-Natural Architectures

While structural optimization improves existing designs, generative morphogenesis creates entirely new architectures inspired by biological principles. AI and ML have triggered a paradigm shift in materials science, moving beyond reliance on explicit physical laws toward data-driven insights. By parsing massive datasets, algorithms identify hidden biological patterns, translating them into actionable engineering logic. Leveraging DL, DRL, and generative models, researchers can explore billions of potential material configurations in a fraction of the time needed by traditional methods. This approach transforms materials science from passive “discovery-by-chance” into active “design-by-intent”. Rather than simple imitation, which is often limited by natural constraints, generative morphogenesis emulates underlying growth principles, such as hierarchical self-organization and multi-functional optimization. Utilizing deep generative models (DGMs), especially GANs and VAES, this field creates novel, synthetic material architectures that retain functional biological “DNA” while exploring unprecedented geometric forms [39,40,41,42,43,44,144,145,146,147,148,149,150].

**Figure 10:**
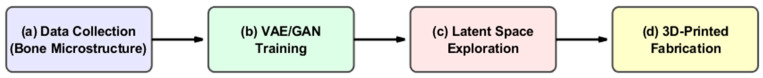
Schematic generative morphogenesis workflow.

Figure 10 illustrates a four-stage generative morphogenesis workflow: (1). Data Generation: Biological patterns, such as bone microstructures, are generated and preprocessed. (2). Model Training: AI/ML models, such as VAEs and GANs, analyze datasets to extract fundamental growth principles and hierarchical self-organization mechanisms. (3). Simulation: Researchers explore “Latent Space” to create synthetic architectures that retain functional biological “DNA” while enhancing engineering performance. (4). Fabrication: Optimized designs are realized through 3D-printing, transitioning from digital concepts to physical structures.

### 4.1. Rise of Generative Architectures: VAEs and GANs

Developing biomimetic materials is challenging due to the need to explore complex, high-dimensional design spaces where hierarchical structures (e.g., bone porosity, wing venation) dictate performance. Traditional optimization techniques, e.g., FEA, are often too slow and iterative. Generative AI models offer efficient, novel solutions to this task:

- **VAEs:** learn to map complex biological morphologies, such as porous bone or cellular materials, into a continuous, low-dimensional “latent space”. Researchers can interpolate within this space to create entirely new designs that go beyond mere replication of the original data while maintaining necessary functional attributes.
- **GANs:** train a generator and discriminator in competition to create realistic and high-fidelity 2D and 3D architectures. This adversarial approach rapidly generates thousands of designs optimized for specific properties, such as enhanced stiffness or energy dissipation capacity. Unsupervised GANs have successfully produced non-natural metamaterials with superior robustness compared to conventional biomimetic benchmarks, such as trabecular bone structures.

This AI-driven approach significantly reduces development times compared to traditional trial-and-error, advancing the field toward rapid prototyping and functional optimization.

### 4.2. Merging Natural DNA with Non-Natural Geometries

Generative morphogenesis merges biological logic with mathematical optimization, allowing AI to translate natural principles into innovative design. By encoding biological structures, such as hierarchical stomata or cellular patterns (e.g., honeycomb and auxetic structures) into an AI system, researchers can explore non-natural geometries overlooked by traditional methods. This process creates optimized, 3D-printable materials with exceptional strength-to-weight ratios.

A prime example is the flectoline façade, a bio-inspired structure mimicking carnivorous plant motion. It utilizes data-driven design to automatically adjust to sunlight without motors, demonstrating a smart, adaptive response. By refining biological efficiencies through AI, these non-natural structures overcome traditional structural limitations, offering both high performance and sustainability.

### 4.3. Key Breakthroughs and Applications

- **Hierarchical Structural Generation:** Advanced 3D-GANs model hierarchical porous materials, generating complex vascular-like networks that surpass traditional design methods by enhancing nutrient transport in tissue engineering scaffolds.
- **Inverse Design and Optimization:** Generative models (GANs/VAEs) enable effective inverse design by automatically creating 3D structures that meet specific target material properties, such as high toughness and low density.
- **Sustainable and Functional Architectures:** Generative tools facilitate the design of complex and performance-optimized porous structures, reducing material consumption while enhancing functionality.

### 4.4. Case Study: Functional Metamaterials Beyond Natural Constraints

Generative morphogenesis creates metamaterials surpassing biological limits by prioritizing functional growth principles over simple mimicry. By decoding the hierarchical logic of natural benchmarks like trabecular bone, AI models like GANs generate synthetic, non-periodic configurations. These AI-driven variants outperform nature, achieving superior performance, such as a 64% increase in modulus or unique negative Poisson’s ratios, effectively translating biological intelligence into engineered high-performance materials [41,42,144,146,151,152,153].

**Figure 11:**
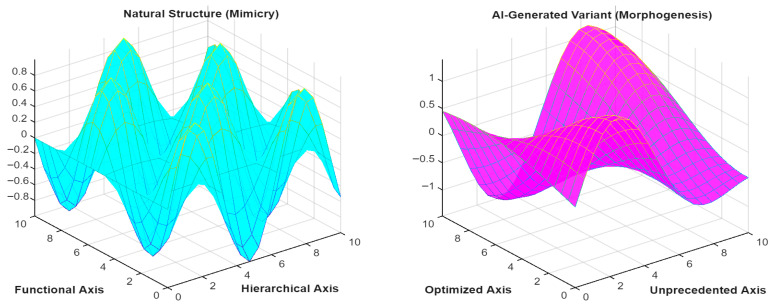
Schematic comparison of structure design paradigms by natural and AI-generated.

Figure 11 compares design paradigms: (Left) Natural, heterogeneous materials, such as bone or sponge, optimized for specific environments; (Right) AI-generated, synthetic metamaterials designed for extreme functionalities, such as high compressibility or negative Poisson’ ratio, that do not exist in nature.

### 4.5. Limitations and Challenges

AI/ML has shifted biomimetic research from imitation of biological forms towards “Generative Morphogenesis”, using Deep Generative Models (e.g., GANs/VAEs) to emulate biological growth mechanisms, such as self-organization for creating non-natural hierarchical architectures. While this enables unprecedented synthetic geometries, the field faces significant bottlenecks: the “black-box” nature of models lacking physical insight, unbuildable generated structures, low design diversity (mode collapse), and a heavy dependency on vast amounts of data. Future progress requires hybrid AI approaches that integrate physical constraints and improve model interpretability [154,155,156,157].

## 5. Adaptive Fabrication: AI-Driven Precision in Biomimetic Fabrication

Despite strong momentum, integrating AI with biomimetic design faces a complexity mismatch: biological systems are stochastic and multi-functional, whereas most current ML models excel only at single-parameter optimization. Consequently, standard neural networks struggle to capture the emergent properties of life. Adaptive fabrication represents a paradigm shift, moving from static manufacturing to dynamic intelligent systems that mimic biological growth by responding to real-time environmental variations.

While standard 3D printing techniques often fail to replicate complex biomimetic hierarchical architectures. AI-driven adaptive manufacturing techniques bridge this gap by integrating in situ sensor data, ML algorithms, and smart materials, such as tissue-like structures and skin-like sensors, into 3D/4D printing workflow [45,46,47,48,49,50,51,52,53,105,158,159,160,161]. 

**Figure 12:**
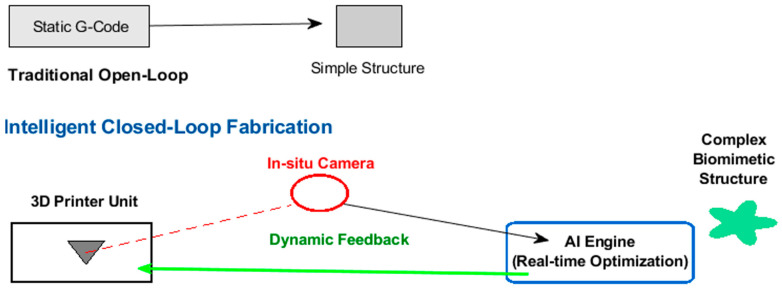
Traditional open-loop and intelligent closed-loop fabrication workflow.

Figure 12 compares traditional open-loop manufacturing processes with intelligent closed-loop manufacturing systems, highlighting AI-driven, real-time in situ monitoring that feeds direct adjustment commands to 3D printing extruders or lasers. Unlike traditional open-loop methods relying on static, preprogrammed G-code, this intelligent closed-loop control dynamically adapts to variations in material and environmental conditions, effectively managing the stochasticity of biological systems. This approach enables the fabrication of complex, heterogeneous biomimetic architectures that are often impossible to produce using standard 3D printing techniques.

### 5.1. Real-Time Monitoring and In Situ Correction

AI models, particularly CV and CNNs, are transforming real-time monitoring of AM processes, such as direct ink writing (DIW) or stereolithography. By comparing in situ imaging data with digital twin models, these AI algorithms instantly detect deviations in structural integrity or feature size. Furthermore, intelligent feedback loops enable on-the-fly adjustments to nozzle velocity, material deposition rates, or light intensity. This proactive control minimizes defects and maximizes the yield of high-fidelity biomimetic scaffold structures [158,162,163,164].

### 5.2. Intelligent Process Parameter Optimization

Due to complex constituents and functional gradients, biomimetic materials require precise control over process parameters, such as temperature, curing time, crosslinking density. AI approaches, specifically Artificial Neural Networks (ANNs) and Genetic Algorithms (GAs), optimize these complex, multi-stimuli-responsive materials by exploring vast parameter spaces inaccessible to manual experimentation, ensuring desired functional and biocompatible characteristics [165,166]. 

**Figure 13:**
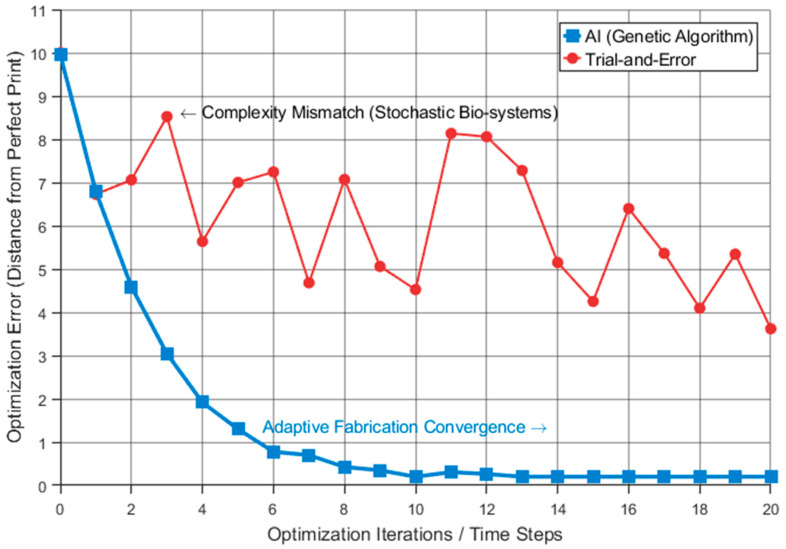
Schematic of machine learning fabrication parameter optimization process.

Figure 13 showcases an ML-based workflow where a GA systematically minimizes objective function errors to determine optimal print speed, viscosity, and temperature, providing more efficient than stochastic, traditional trial-and-error methods.

### 5.3. Integrating Smart Materials in AI-Driven 3D/4D Printing

Integrating smart materials into 3D and 4D workflows elevates biomimetic fabrication from inert assembly to creating active, living architectures with sensing and actuation capabilities. AI optimizes material distributions to mimic biological anisotropy, programming stimulus-responsive polymers and nanocomposites for controlled temporal transformations, achieving autonomous shape-morphing and self-healing [161,167,168,169,170,171,172,173,174].

The key advancements in smart fabrication include:

- AI-Optimized Design: AI navigates complex design spaces for stimulus-responsive materials, enabling tailored temporal responses that mimic biological anisotropy.
- Embedded Functionality: Smart materials, including tissue-like structures, skin-like sensors, shape-memory polymers, hydrogels, and liquid crystal elastomers, are seamlessly embedded directly into hierarchical designs and printing morphology.
- Controlled Adaptability: AI-optimized material gradients enable structures to undergo controlled temporal transformations in response to environmental fluctuations, facilitating adaptive, emergent behaviors of natural organisms.

### 5.4. Closed-Loop Manufacturing and 4D Printing in Adaptive Fabrication

To achieve a biomimetic paradigm shift requires evolving from linear fabrication to a closed-loop manufacturing ecosystem. In this framework, biological sensors act as a nervous system, providing real-time data on material flow, thermal gradients, and structural integrity. AI then instantly adjusts printing parameters, such as nozzle velocity or laser intensity, to compensate for material stochasticity, ensuring high-fidelity results that mirror natural hierarchical complexity [175,176].

Building on this adaptive foundation, 4D printing introduces the dimension of time, utilizing smart stimuli-responsive materials that alter their shape, mechanical properties, or functional state post-production when exposed to external triggers like heat, humidity, pH, or light. Unlike static 3D objects, 4D-printed architectures exhibit emergent behavior, allowing them to self-assemble or adapt to their environment [177,178,179,180,181].

Key Examples of AI-Driven 4D Biomimicry:

- Self-Morphing Soft Robotics: AI-optimized 4D printing arranges hygroscopic (water-responsive) fibers to create motor-free actuators that mimic plant-like movements, navigating complex environments by changing shape in response to moisture.
- Adaptive Biomedical Scaffolds: AI-enhanced 4D printing enables the creation of vascular stents or bone scaffolds that remain compressed for minimally invasive insertion, then expand to a precise, bio-inspired geometry upon reaching body temperature. By integrating real-time sensor data during the print, AI ensures the material’s “shape memory” is tuned to the specific irregularities of a patient’s anatomy [53,182].

### 5.5. Overcoming the Challenge of Data Scarcity Through Active Learning

In AI-driven manufacturing, the scarcity of material-specific and large-scale datasets poses a significant barrier. Active Learning (AL) strategies are increasingly adopted to overcome this, as they can identify the most informative experiments to execute. By iteratively updating the underlying models, AL optimizes printing formulations with remarkably few trials. This capability is vital for developing novel, sustainable materials, where traditional approaches are expensive and time-consuming [52,164,183].

## 6. Data-Driven Discovery Platforms: Integrating Biomimetics Research

Biomimetics is shifting from traditional trial-and-error methods to data-driven closed-loop autonomous discovery labs where AI models propose, synthesize, and test materials with minimal human intervention [184,185,186]. While the conceptual framework is robust, leveraging ecosystems like MP and pyiron to synchronize digital and physical workflows, the current maturity level remains largely in the “prototyping and pilot” phase. Fully integrated systems exist in high-end academic and industrial labs (e.g., for thin films or simple polymers), but most biomimetic applications, such as complex hierarchical lattices or gradient polymers, are still transitioning from theoretical models to fully automated execution. Practical constraints are significant: the high capital cost of robotic hardware and the fabrication limits of current 3D printing and synthesis platforms often struggle with the multi-scale precision required for biological mimicry. Data integration remains a bottleneck, as standardizing messy, diverse biological datasets into machine-readable formats is essential for ML models to accurately predict properties like mechanical performance or degradation rates in biomimetic polymers and tissue scaffolds [187,188,189,190,191,192]. Furthermore, by integrating protein engineering for biosensors and nanoscale neuromorphic devices for enhanced perception, these platforms now facilitate the fully autonomous synthesis and optimization of next-generation materials [193,194,195,196,197,198,199,200,201].

**Figure 14:**
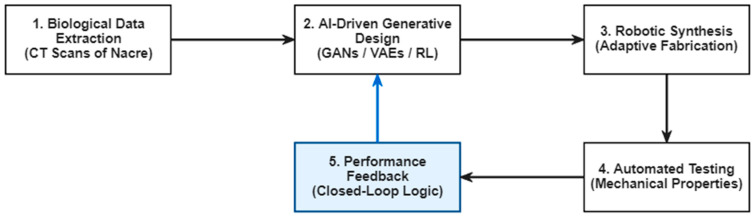
Schematic diagram of an autonomous closed-loop discovery platform in biomimetics.

As illustrated in Figure 14, these platforms successfully integrate biological data extraction (e.g., CT scans of nacre), generative design, and robotic synthesis. However, while “closing the loop” with automated testing is technically feasible for specific properties like mechanical stiffness or degradation rates, achieving universal autonomy across complex chemical spaces remains a significant engineering challenge. A summary of advantages and limitations of various AI architectures in typical biomimetic applications is listed in Table A1 in Appendix A.

The shift toward AI-driven discovery faces several challenges, starting with the limited quality and volume of available data. The “black box” nature of AI also creates an interpretability crisis. The practical constraints for integrating digital designs with physical fabrication remains difficult, as manufacturing constraints (e.g., 3D printing resolution, cost, fabrication limits, data integration) often clash with deploying AI outputs. While data-driven platforms are beginning to bridge these gaps, we must solve issues regarding data scarcity, quality, interpretability, and synthesizability to accelerate the discovery of functional biomimetic materials.

### 6.1. The Challenges of Data Scarcity and Quality

A major bottleneck for these platforms is the vast data availability gap between materials science and traditional AI. While CV and NLP utilize massive public databases such as ImageNet, biomimetic research is hindered by fragmented, non-standardized data often confines to proprietary silos [6].

To bridge this gap, the implementation of data standardization strategies, such as unified ontologies and FAIR (findable, Accessible, Interoperable, Reusable) data principles, is essential to harmonize disparate morphological and mechanical descriptors. Furthermore, the development of specialized benchmark datasets for biological microstructures, such as structural details of mineralized collagen fibrils or seed pod mechanics, is critical for objective model comparison. Currently, these high-fidelity datasets are expensive and slow to produce, leading to a “small-data” problem where models frequently overfit, capturing noise rather than fundamental physical laws. Consequently, high benchmark scores often fail to translate to functional outcomes. Addressing this requires rigorous experimental validation, including 3D printing and mechanical testing of AI-generated designs, to ensure that computational outputs align with real-world physical performance and structural integrity [202,203,204,205].

### 6.2. The Interpretability Crisis and XAI

Another major challenge in deploying advanced DL is the “black box” nature of these algorithms. In safety-critical fields, such as aerospace and tissue engineering, understanding the rationale behind an AI recommendation is as crucial as its performance [206]. For example, when creating biomimetic dental implants, engineers must ensure the AI’s logic adheres to physical biomechanical principles for long-term biocompatibility. This “interpretability crisis” creates a significant trust gap, causing engineers and clinicians to hesitate in adopting AI-driven, opaque methods.

To bridge this gap, the integration of XAI and Physics-Informed Neural Networks (PINNs) is necessary. These approaches embed established scientific laws into the learning process to ensure interpretable results. Without XAI, researchers remain reluctant to adopt designs lacking explicit physical rationale. Consequently, emerging platforms are now integrating symbolic regression and PINNs to ensure outputs are not only high-performing but also physically consistent and human-interpretable [207].

**Figure 15:**
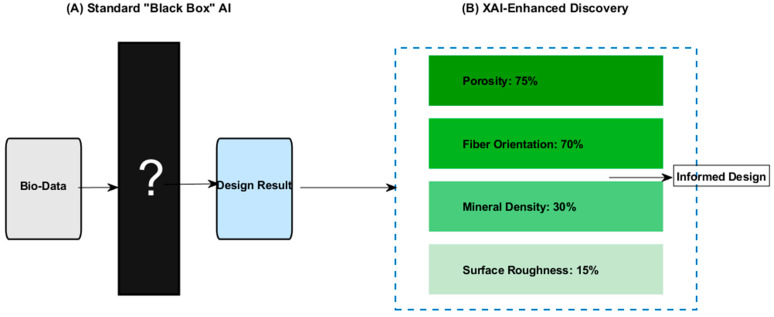
Schematic interpretability gap.

Figure 15 illustrates the interpretability gap by comparing a standard “Black Box” model to an XAI-enhanced model. In the diagram, the black box is depicted as a mystery, represented by a dark block and a question mark. In contrast, the XAI model uses a heatmap to rank which biological features, such as porosity and fiber orientation, most heavily influence the final design recommendations.

### 6.3. Bridging the Digital-Physical Gap

A significant obstacle from practical constraints remains in integrating AI-driven design with physical fabrication: manufacturability.

- Fabrication Limits: While AI can identify ideal, high-performance structural lattices, e.g., bone-inspired designs, and nanoscale protein engineering for biosensors. These patterns often exceed the resolution limits or material constraints of current 3D printing and bio-fabrication techniques for multi-scale complexity of biological structures. Consequently, a substantial gap exists between “in-silico” digital optimization and physical, “in-vivo” performance.
- Data integration: Standardizing diverse datasets from disparate sources (HT simulations vs. physical experiments) remains difficult, requiring sophisticated middleware to ensure ML models can accurately predict performance.
- Cost and Complexity: The high capital investment required for specialized robotics and the computational overhead of real-time AI optimization currently restrict these labs to well-funded research institutions and specific high-value materials, such as neuromorphic devices to tissue scaffolds.

To close this loop, manufacturing constraints, such as toolpath limitations, printing resolution, curing times, material viscosity, and cooling rates, must be incorporated directly into the AI’s loss function, in addition to considering other practical constraints. Only by bridging this divide between the digital and the physical can design platforms move beyond theoretical optimization to enable reliable, next-generation biomimetic material manufacturing [208,209,210].

### 6.4. Federated Learning: Decentralized Intelligence and Privacy

While centralized platforms unify data, federated learning (FL) addresses the privacy and security hurdles of data sharing by allowing AI models to be trained across decentralized machine learning; models are trained locally across multiple institutions, and only updated parameters are aggregated, preventing raw data transfer. This approach is essential when sensitive information or proprietary data restricts centralized pooling. However, FL is constrained by high communication costs, technical complexity in managing heterogeneous datasets across clients, and potential security vulnerabilities, such as information leakage through exchanged model updates. Crucially, current FL implementations for data-driven discovery do not cover specialized domains like soft robotics or biohybrid systems, nor do they fully address the complex ethical implications of autonomous design decisions [211,212,213,214,215,216,217].

## 7. Conclusions and Future Perspectives

Integrating AI into biomimetics transitions research from passive observation to proactive, data-driven closed-loop predictive engineering revolutionizing tissue engineering and sustainable materials [218]. Future progress depends on overcoming a “complexity mismatch” where high-dimensional biological systems surpass current computational model capabilities, and developing transparent AI, as current tools often lack interpretability for safety-critical applications [219,220].

Key future research directions [221,222,223,224,225]:

- Explainable AI (XAI) Development: Creating interpretable AI models that provide biologically justified designs, crucial for structural safety and clinical reliability.
- Multi-Modal Data Mining: Developing techniques to synthesize heterogeneous datasets—imaging, genomics, and clinical data to better emulate complex life-like systems.
- Autonomous “Self-Driving” Laboratories: Integrating AI-driven design with robotic synthesis to minimize human bias and accelerate discovery cycles.
- Neuromorphic AI Architectures: Leveraging biological principles to design energy-efficient, nature-inspired computing systems.

## Figures and Tables

**Table 1:** Performance quantitative comparison: traditional vs. AI-driven topology optimization.

Metric	Traditional TO (e.g., SIMP)	AI-Driven TO (e.g., CNNs, GANs)
Computational Speed	Slow; requires hundreds of iterative FEA simulations	Fast; surrogate models predict designs almost instantly after training.
Speedup Factor	Baseline (1×)	Up to 30× faster than traditional TO.
Accuracy	High (exact physics calculation)	High (*R*^2^ > 0.95 for regression models) but may require validation.
Design Cycle Time	Months to years [Prompt]	Days to weeks [Prompt]
Material Reduction	Up to 30–60%	Up to 70% in specific aerospace applications.

## Data Availability

No new data were created or analyzed in this study. Data sharing is not applicable to this article.

## Abbreviations

The following abbreviations are used in this manuscript:

AI	Artificial Intelligence
AL	Active Learning
AM	Additive Manufacturing
ANNs	Artificial Neural Networks
BCDB	Block Copolymer Phase Behavior Database
CFD	Computational Fluid Dynamics
CNNs	Convolutional Neural Networks
CV	Computer Vision
DFT	Density Functional Theory
DGMs	Deep Generative Models
DL	Deep Learning
DRL	Deep Reinforcement Learning
FEA	Finite Element Analysis
GANs	Generative Adversarial Networks
GNNs	Graph Neural Networks
HT	High-Throughput
MD	Molecular Dynamics
ML	Machine Learning
MP	Materials Project
NLP	Natural Language Processing
PCA	Principal Component Analysis
PINNs	Physics-Informed Neural Networks
SEM	Scanning Electron Microscopy
TEM	Transmission Electron Microscopy
TO	Topology Optimization
t-SNE	t-distributed Stochastic Neighbor Embedding
U-Net	U-shaped Convolutional Network
VAEs	Variational Autoencoders
XAI	Explainable AI
μCT	Micro-Computed Tomograph

## Appendix A

The following table summarizes AI architectures used to translate biological intelligence into engineering logic, facilitating rapid exploration of design spaces in biomimetics [9,41,147,226,227,228,229,230,231,232,233].

**Table A1:** Summary of AI architectures in biomimetic applications.

**AI Architectures**	**Typical Biomimetic Applications**	**Advantages**	**Limitations**
**CNNs** (Convolutional Neural Networks)	Pattern recognition in biological structures (e.g., bone microtomography), feature extraction from microscopic images.	Automatic feature engineering; high accuracy in image-based pattern recognition.	“Black box” nature (low interpretability); requires large labeled datasets; risk of overfitting.
**GANs** (Generative Adversarial Networks)	Generating novel nature-inspired geometries (e.g., optimizing façade patterns); reconstructing 3D forms from 2D images.	High-fidelity, realistic image generation; capable of synthesizing novel, complex structures.	Prone to mode collapse (limited output diversity); difficult to train and control.
**VAEs** (Variational Autoencoders)	Generative morphogenesis; mapping biological structures into latent space for controlled design generation.	Stable training; structured latent space allows for better control over generated output.	Generated images can be blurry; less realistic than GAN outputs.
**DRL** (Deep Reinforcement Learning)	Adaptive fabrication control; autonomous agent decision-making for structural growth or robotic movement.	High adaptability; capable of continuous learning and optimizing sequential decisions.	Unstable training process; requires significant computational resources and data.
**GNNs** (Graph Neural Networks)	Modeling hierarchical biological networks (e.g., protein interactions); analyzing structures without Euclidean grid representation.	Natural representation of graph-structured biological data; captures hidden topological information.	Scalability issues with very large graphs; complex to interpret.
**PINNs** (Physics-Informed Neural Networks)	Structural optimization constrained by physical laws; rapid simulation of material behavior in tissue engineering.	Improved generalization with small datasets; integrates physical constraints into the learning process.	High computational cost for training; optimization can be difficult to converge.
